# The anti-inflammatory activity of GABA-enriched *Moringa oleifera* leaves produced by fermentation with *Lactobacillus plantarum LK-1*

**DOI:** 10.3389/fnut.2023.1093036

**Published:** 2023-03-09

**Authors:** Long Zheng, Xuli Lu, Shengtao Yang, Ying Zou, Fanke Zeng, Shaohao Xiong, Yupo Cao, Wei Zhou

**Affiliations:** Key Laboratory of Tropical Crop Products Processing of Ministry of Agriculture and Rural Affairs, Agricultural Products Processing Research Institute, Chinese Academy of Tropical Agricultural Sciences, Zhanjiang, Guangdong, China

**Keywords:** *Moringa oleifera* leaves, fermentation, gamma-aminobutyric acid, anti-inflammatory activity, RAW 264.7 cells

## Abstract

**Introduction:**

Gamma-aminobutyric acid (GABA), one of the main active components in *Moringa oleifera* leaves, can be widely used to treat multiple diseases including inflammation.

**Methods:**

In this study, the anti-inflammatory activity and the underlying anti-inflammatory mechanism of the GABA-enriched *Moringa oleifera* leaves fermentation broth (MLFB) were investigated on lipopolysaccharide (LPS)-induced RAW 264.7 cells model. The key active components changes like total flavonoids, total polyphenols and organic acid in the fermentation broth after fermentation was also analyzed.

**Results:**

ELISA, RT-qPCR and Western blot results indicated that MLFB could dose-dependently inhibit the secretions and intracellular expression levels of pro-inflammatory cytokines like 1β (IL-1β), interleukin-6 (IL-6), interleukin-8 (IL-8) and tumor necrosis factor-α (TNF-α). Furthermore, MLFB also suppressed the expressions of prostaglandin E_2_ (PGE_2_) and inducible nitric oxide synthase (iNOS). Moreover, the mRNA expressions of the key molecules like Toll-like receptor 4 (TLR-4) and nuclear factor (NF)-κB in the NF-κB signaling pathway were also restrained by MLFB in a dose-dependent manner. Besides, the key active components analysis result showed that the GABA, total polyphenols, and most organic acids like pyruvic acid, lactic acid as well as acetic acid were increased obviously after fermentation. The total flavonoids content in MLFB was still remained to be 32 mg/L though a downtrend was presented after fermentation.

**Discussion:**

Our results indicated that the MLFB could effectively alleviate LPS-induced inflammatory response by inhibiting the secretions of pro-inflammatory cytokines and its underlying mechanism might be associated with the inhibition of TLR-4/NF-κB inflammatory signaling pathway activation. The anti-inflammatory activity of MLFB might related to the relative high contents of GABA as well as other active constituents such as flavonoids, phenolics and organic acids in MLFB. Our study provides the theoretical basis for applying GABA-enriched *Moringa oleifera* leaves as a functional food ingredient in the precaution and treatment of chronic inflammatory diseases.

## 1. Introduction

Inflammation is a natural immunological response that the immune system reacted to outside stimulus like pathogen (1). However, the inordinate and uncontrolled inflammation will also lead to apoptosis of immune cells and immunologic derangement which resulting in the chronic degenerative diseases and cancer (2, 3). Although various steroids and non-steroidal anti-inflammatory drugs (NSAIDs) like azathioprine and aspirins have been used to treat these acute and chronic inflammatory diseases, long-term use of these drugs may also lead to various adverse effects to human health (1). Therefore, the development of natural anti-inflammatory drugs with greater efficacy and minimal toxicity has attracted more and more attention. Besides, with the improvement of consumption level, natural anti-inflammatory functional foods are more and more popular in humans (4).

Gamma-aminobutyric acid (GABA) is a non-protein amino acid that can be found in plants, bacteria and animals (5). It's an important inhibitory neurotransmitter that presented in mammal central nervous system with multiple physiological functions including anti-inflammation (6). For example, GABA showed obvious anti-inflammatory effect on streptozotocin-treated mice by decreasing the synthesis of inflammatory mediators like IL-1β, TNF-α, IFN-γ, IL-12, and increasing the production of anti-inflammatory mediator TGF-β1 (7). Besides, GABA restrained the expression of inflammatory cytokines (L-6, IL8, and TNF-α) and alleviated inflammatory response of MAC-T cells induced by LPS *via* the TLR4-MyD88-NFκB signaling pathway (8). Except for anti-inflammation, GABA has also been reported to possess hypotensive, antidiabetic, immunity enhancement, and sedative effects, and has been applied to alleviate sleeplessness, depression and improve visual cortical function (6, 9). Despite its various physiological functions, GABA content in natural animal- and plant-based food products is pretty low which cannot meet people's needs (6). Therefore, GABA enrichment has attracted increasing attention and a variety of related functional foods have been developed like GABA-enriched tea, soft sweets, beverages, dairy products and so on. At present, microbial fermentation is the most common method to enrich GABA because of its ability to produce lactic acid, flavonoids, polyphenols and other active substances beneficial to human health (5, 10). Up to now, microbial fermentation has been widely applied in the GABA enrichment in strawberry juice (6), green tea (11), water dropwort (10), etc.

*Moringa oleifera*, also known as “drumstick tree” or simply Moringa in English, is a perennial deciduous tropical plant with a variety of bioactive compounds (12). Because of its nutritional and medicinal value, *Moringa oleifera* has been regarded as one of the most economically valuable plants and is widely used in food, industry, agriculture and medicine in the developing countries (13). *Moringa oleifera* leaves contain multiple active constituents like protein, amino acids, polysaccharides, dietary fiber, phenols, flavonoids, phytosterols, glycosides (2), and are reported to possess multiple pharmacological activities like antioxidant, anti-inflammatory, anti-hypertensive, hypoglycemic, hypolipidemic, liver and kidney protective, as well as anti-cancer effects (14–16). Previous studies have found that GABA as well as other active constituents like flavonoids, polyphenols, most amino acids, oligosaccharides, organic acids, nucleosides of *Moringa oleifera* leaves are significantly enhanced after fermentation (12, 17), which suggested that *Moringa oleifera* leaves were ideal candidate for GABA enrichment. Therefore, GABA enrichment in *Moringa oleifera* leaves may provide new idea for its application in functional foods. However, to our knowledge, there are still no studies on the enrichment of GABA using *Moringa oleifera* leaves as raw material. As a consequence, we tried *Lactobacillus* fermentation to enrich GABA in *Moringa oleifera* leaves and the result showed that the GABA content of the fermented *Moringa oleifera* liquid increased to 209 mg/L under the optimal fermentation conditions, which was 1.45 times higher than that of the unfermented (18). However, the other key active components changes in the fermentation broth after fermentation has not been further studied. Besides, the anti-inflammatory activity of the fermentation broth and the underlying anti-inflammatory mechanism is still unclear, which greatly limited its application in functional foods.

Therefore, in order to exploit the application value of GABA-enriched *Moringa oleifera* leaves fermentation broth (MLFB) in functional foods, the key active components changes like total flavonoids, total polyphenols and organic acid in the fermentation broth after fermentation was analyzed, and the anti-inflammatory activity as well as the underlying anti-inflammatory mechanism of the fermentation broth were investigated on LPS-induced RAW 264.7 cells model. This study may provide theoretical basis for the application of MLFB on the anti-inflammatory functional foods.

## 2. Materials and methods

### 2.1. Materials

*Moringa oleifera* leaves powder was provided by Henan Jinlamu Bio-technology (Hebi, Henan, China). Lipopolysaccharide (LPS), 3-(4,5-dimethylthiazol-2-yl)-2,5-diphenyltetrazolium bromide (MTT), Dulbecco's modified Eagle medium (DMEM) and dimethyl sulfoxide (DMSO) were from Solarbio Science and Technology (Beijing, China). *Lactobacillus plantarum LK-1* was isolated from pickles in our laboratory (19). Fetal bovine serum (FBS) and ethylene diamine tetraacetic acid (EDTA) digestive juice were from Gibco (Carlsbad, CA, USA). IL-6, IL-8, IL-1β, TNF-α detection kits (enzyme-linked immunosorbent assay) were from Nanjing Jiancheng Biotechnology (Nanjing, Jiangsu, China). Nucleic Acid Stain was from Beijing Dingguo Changsheng Biotechnology (Beijing, China). Reverse transcription kit and fluorescent dye kit were from Guangzhou Jisai Biotechnology (Guangzhou, Guangdong, China).

### 2.2. Preparation of MLFB and its key active components analysis before and after fermentation

GABA-enriched *Moringa oleifera* leaves fermentation broth was obtained using *Lactobacillus plantarum LK-1* as fermentation strain according to the previous study (18). In detail, the *Moringa oleifera* leaves powder was added to distilled water at 1:25 (w/w), 3% (w/w) of glucose was added and monosodium glutamate was then added at the final concentration of 5 g/L. After autoclaving at 105°C for 20 min, the *Moringa oleifera* solution was fermented with *Lactobacillus plantarum LK-1* at 35°C for 72 h. After that, the fermentation mixture was centrifuged at 6,000 *g* for 30 min, then the supernatant was collected and named as MLFB for the following anti-inflammatory activity evaluation. The GABA content of MLFB was detected to be 209 mg/L by high performance liquid chromatography (HPLC) with pre-column o-phthaldialdehyde (OPA) derivatization (18). The result of GABA content analysis in MLFB and *Moringa oleifera* leaves solution (MLS) along with their HPLC spectrograms can be seen in the Appendix. The total flavonoids, total polyphenols and organic acid content in MLFB and MLS were detected according to the methods of Li et al. (17).

### 2.3. Cell culture

The macrophages RAW 264.7 were provided by the Procell Life Technology (Wuhan, Hubei, China). The cells were cultured in DMEM with 10% FBS at 37°C in an atmosphere of 5% CO_2_, as the cell provider required.

### 2.4. Cell viability assay

The cell viability was determined by MTT assay (2). Cells in logarithmic phase was dispersed in DMEM containing 10% FBS. Then the cell suspension was evenly transferred to 96-well plates at 5 × 10^3^ cells/well. After cultivating for 12 h, the culture medium was removed and 100 μL fresh culture medium with different concentrations of MLFB (31.25, 62.5, 125, 250 and 500 μg/mL) was added to each well. 12 h later, 10 μL 5 mg/mL MTT was added to each well, and the supernatant was gently removed after 3 h of culture. Then 150 μL DMSO was added to each well and the mixture was oscillated for 10 min. The absorbance of the mixture was detected at 570 nm using a microplate reader (Bio-Tek, Winooski, VT, USA). The relative activity of RAW 264.7 cells was calculated considering the activity of cells treated without MLFB as 100%.

### 2.5. Enzyme-linked immunosorbent assay (ELISA)

The concentrations of inflammatory factors (IL-1β, IL-6, IL-8 and TNF-α) in cell supernatants were detected using ELISA kits referring to the manufacturer's descriptions. RAW 264.7 cells suspension (1 × 10^5^ cells/mL) was inoculated into 12-well plate at 1 × 10^5^ cells/well. After cultivating for 12 h, the supernatant in each well was removed and the cells were treated with different concentrations of MLFB (125, 250, 500 μg/mL) for 1 h. After incubating with 1 μg/mL LPS for 24 h, the mixture was collected and centrifuged for 20 min at 3,000 *g*. The secretion levels of IL-1β, IL-6, IL-8, and TNF-α in supernatants of each group were measured and the standard curves of these inflammatory factors were made referring to the manufacturer's descriptions in ELISA kits.

### 2.6. Real-time reverse transcription quantitative polymerase chain reaction

After RAW 264.7 cells were treated with LPS and different concentrations of MLFB for 24 h, the mRNA expressions of IL-1β, IL-6, TNF-α, NF-κB, PGE_2_, TLR-4 in RAW 264.7 cells were determined by RT-qPCR (6). Firstly, TRIzol reagent was used to extract the total RNA of cells. Then RNA was synthesized into cDNA by cDNA reverse transcription kit referring to the manufacturer's descriptions. Finally, the RT-qPCR was proceeded in 20 μL reaction system containing 2 μL cDNA, 1 μL primer pairs (10 μmol/L), 10 μL SYBR Green PCR Master Mix, 0.4 μL 50 × ROX Reference Dye 2 and 6.6 μL ultra-pure distilled water. The PCR conditions were as followings: initial denaturation at 95°C for 5 min, 40 cycles at 95°C for 10 s, 60°C for 34 s, and the melting curve was obtained at 95°C for 15 s, 60°C for 1 min and 95°C for 15 s in the ABI 7500 real-time fluorescent quantitative PCR System (Applied Biosystems, Foster, CA, USA). β-actin was used as internal control and the primers used here are shown in Table 1.

**Table 1:** Primer sequences of RT-qPCR.

**Primer name**	**Primer sequence**	**Product size/bp**
β-actin	Forward: 5′-GCTTCTAGGCGGACTGTTAC-3′	100
	Reverse: 5′-CCATGCCAATGTTGTCTCTT-3′	
IL-1β	Forward: 5′-GTGTCTTTCCCGTGGACCTT-3′	121
	Reverse: 5′-CGTCACACACCAGCAGGTTA-3′	
IL-6	Forward: 5′-CCACTTCACAAGTCGGAGGC-3′	117
	Reverse: 5′-TTTCTGCAAGTGCATCATCGTT-3′	
NF-κB	Forward: 5′-ACACCTCTGCATATAGCGGC-3′	152
	Reverse: 5′-GGCACCACTCCCTCATCTTC-3′
PGE_2_	Forward: 5′-CACCTTCGCCATATGCTCCT-3′	154
	Reverse: 5′-GACCGGTGGCCTAAGTATGG-3′	
TLR-4	Forward: 5′-AGATCTGAGCTTCAACCCCTTG-3′	137
	Reverse: 5′-AGAGGTGGTGTAAGCCATGC-3′	
TNF-α	Forward: 5′-CCACCACGCTCTTCTGTCTA-3′	105
	Reverse: 5′-TGAGGGTCTGGGCCATAGAA-3′	

### 2.7. Western blot

After treated with LPS and different concentrations of MLFB for 24 h, the protein expression levels of IL-1β, IL-6, TNF-α and iNOS in RAW 264.7 cells were determined by Western blot (20). Firstly, the cells were lysed by 400 μL cell lysis buffer containing protease inhibitors. Then the lysate was stirred on ice for 30 min and transferred to a 100°C water path for 10 min. After centrifuging the lysate at 6,000 *g* for 6 min at 4°C, the supernatant was gathered and the protein content was determined by a BCA protein assay kit (Solarbio Science and Technology Crop., Beijing, China). Then the proteins were analyzed by 10% SDS-PAGE and transferred to a PVDF membrane. After blocking with 5% skimmed milk for 60 min, the PVDF membrane was incubated with the primary antibody at 4°C for a night. Then the PVDF membrane was washed with TBST (50 mmol/L Tris-HCl, 150 mmol/L NaCl and 0.1% v/v Tween-20) for three times before incubating with the secondary antibody for 40 min at room temperature. The bands on the membrane were quantified by an automatic gel imaging analysis system (Peiqing science and technology Crop., Shanghai, China). β-actin was used as the internal control, and the results were displayed referring to the control.

### 2.8. Statistical analysis

All tests were performed at least in triplicate and the experimental results were presented as mean ± standard deviation (SD). SPSS Version 17.0 (SPSS Inc., Chicago, IL) was used to analyze the significant differences between samples and *P* < 0.05 was regarded as statistically significant. All the figures were drawn by origin 8.0 (OriginLab Corp., Northampton, USA).

## 3. Results and discussion

### 3.1. Cytotoxicity of MLFB on RAW 264.7 cells

**Figure 1:**
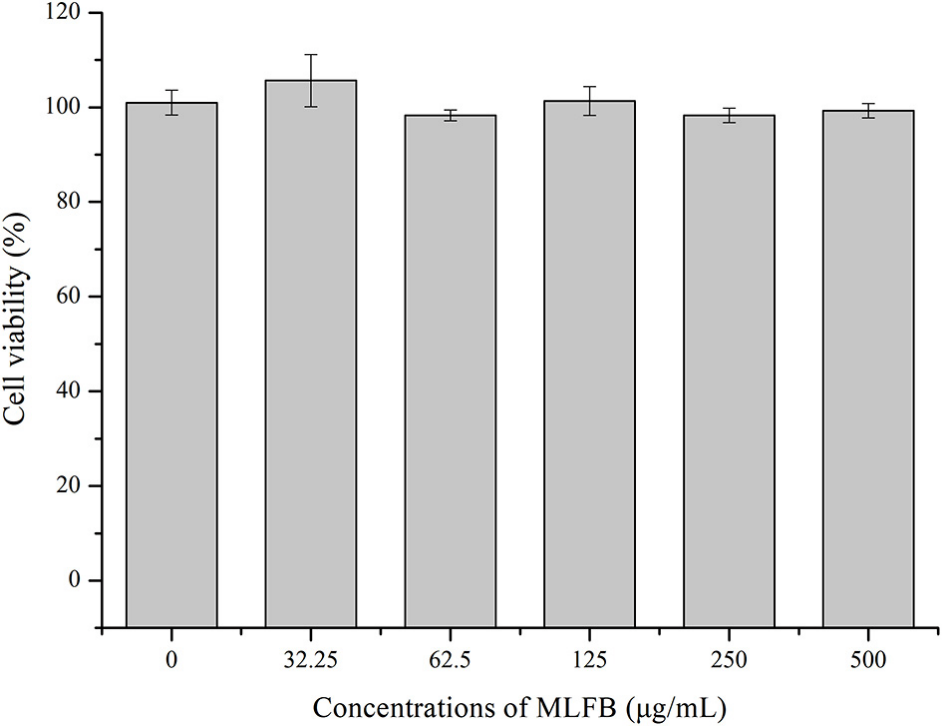
Effects of MLFB on the viability of RAW 264.7 cells.

The cytotoxic effect of MLFB on RAW 264.7 cells was determined by MTT assay. As shown in Figure 1, after treated with different concentrations of MLFB, the viability of RAW 264.7 cells showed no significant changes (*P* > 0.05). After treated with 500 μg/ml MLFB for 12 h, the cell viability was measured to be 99.33%. The result indicated that the MLFB with concentration under 500 μg/mL had no obvious toxicity to cells. The result was consistent with the study result of Lin et al. (20) who stated that the brown seaweed *Laminaria japonica* fermented by *Bacillus subtilis* also showed no significant cytotoxic effects on RAW 264.7 cells. According to the result of cytotoxic effect, concentrations of 125, 250 and 500 μg/mL of MLFB were selected as the standard test concentrations in the follow-up experiments.

### 3.2. MLFB inhibits the productions and expressions of pro-inflammatory cytokines in LPS-induced RAW 264.7 cells

**Figure 2:**
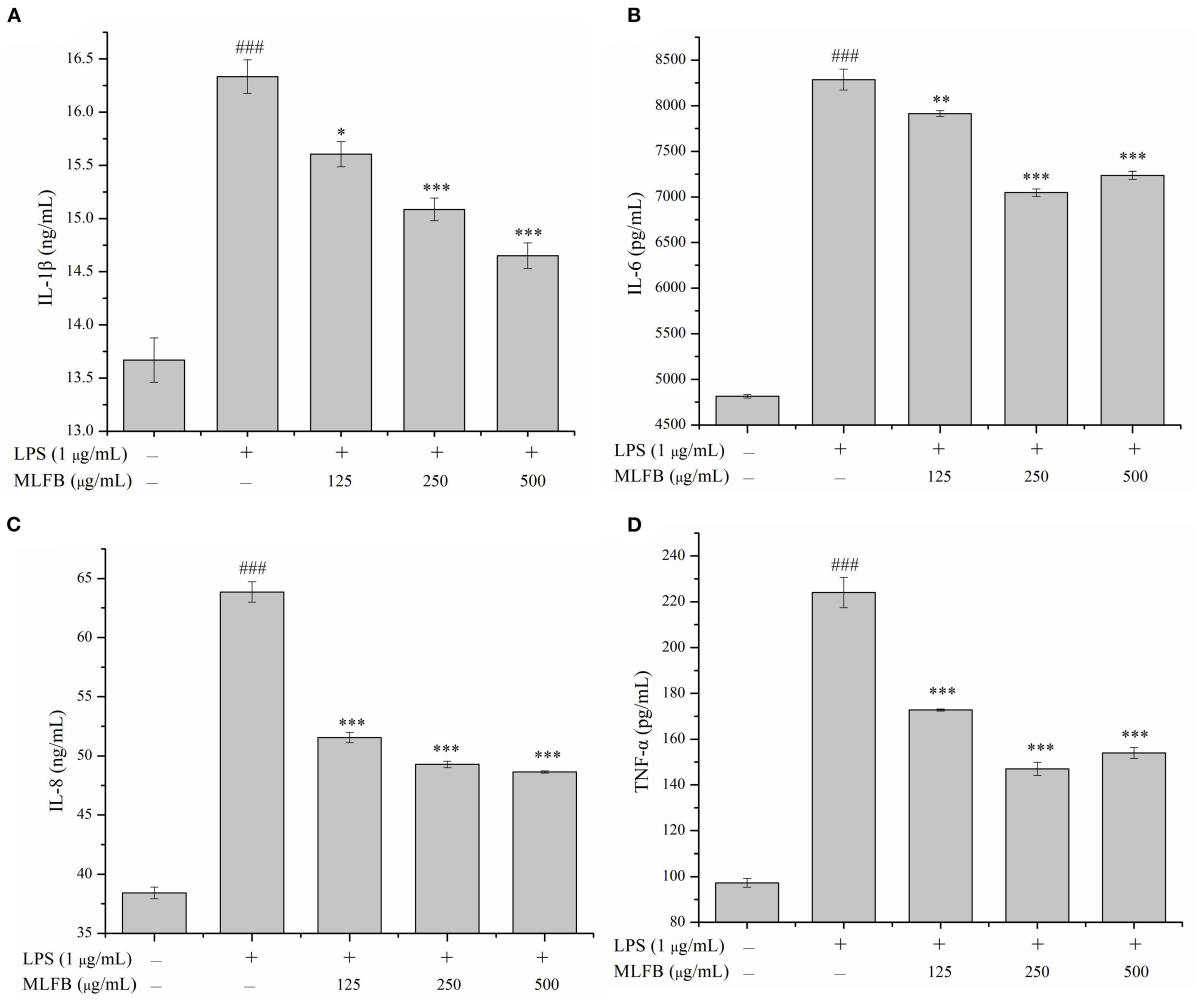
Effects of MLFB on the secretions of IL-1β **(A)**, IL-6 **(B)**, IL-8 **(C)**, and TNF-α **(D)** in LPS-induced RAW 264.7 cells. ^#^Means statistical difference compared with blank control group (^###^*P* < 0.001). *Means statistical difference compared with LPS-induced inflammatory model group (*P < 0.05, **P < 0.01, ***P < 0.001).

**Figure 3:**
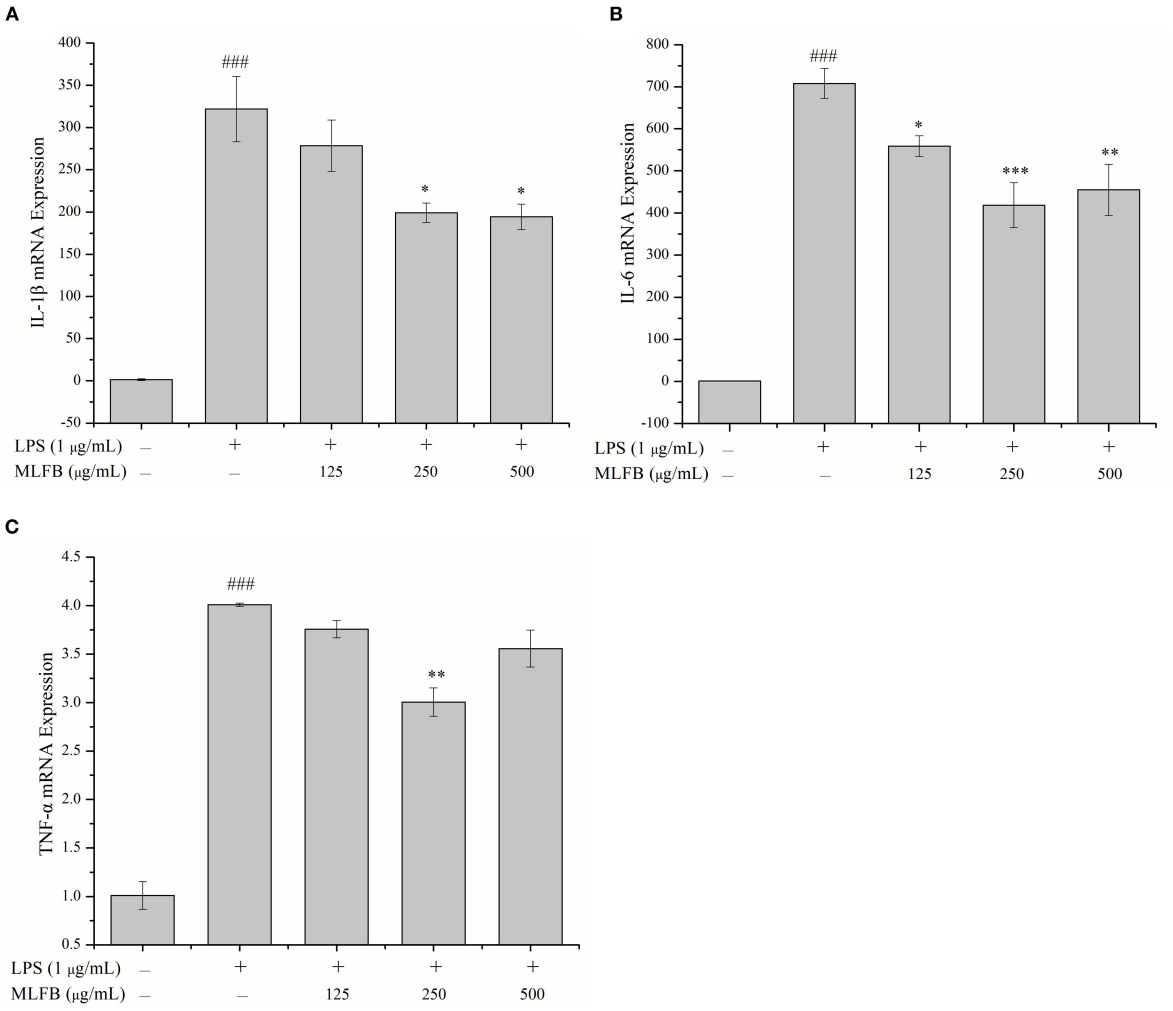
Effects of MLFB on the mRNA expressions of IL-1β **(A)**, IL-6 **(B)**, TNF-α **(C)** in LPS-induced RAW 264.7 cells. ^#^Means statistical difference compared with blank control group (^###^*P* < 0.001). *Means statistical difference compared with LPS-induced inflammatory model group (*P < 0.05, **P < 0.01, ***P < 0.001).

**Figure 4:**
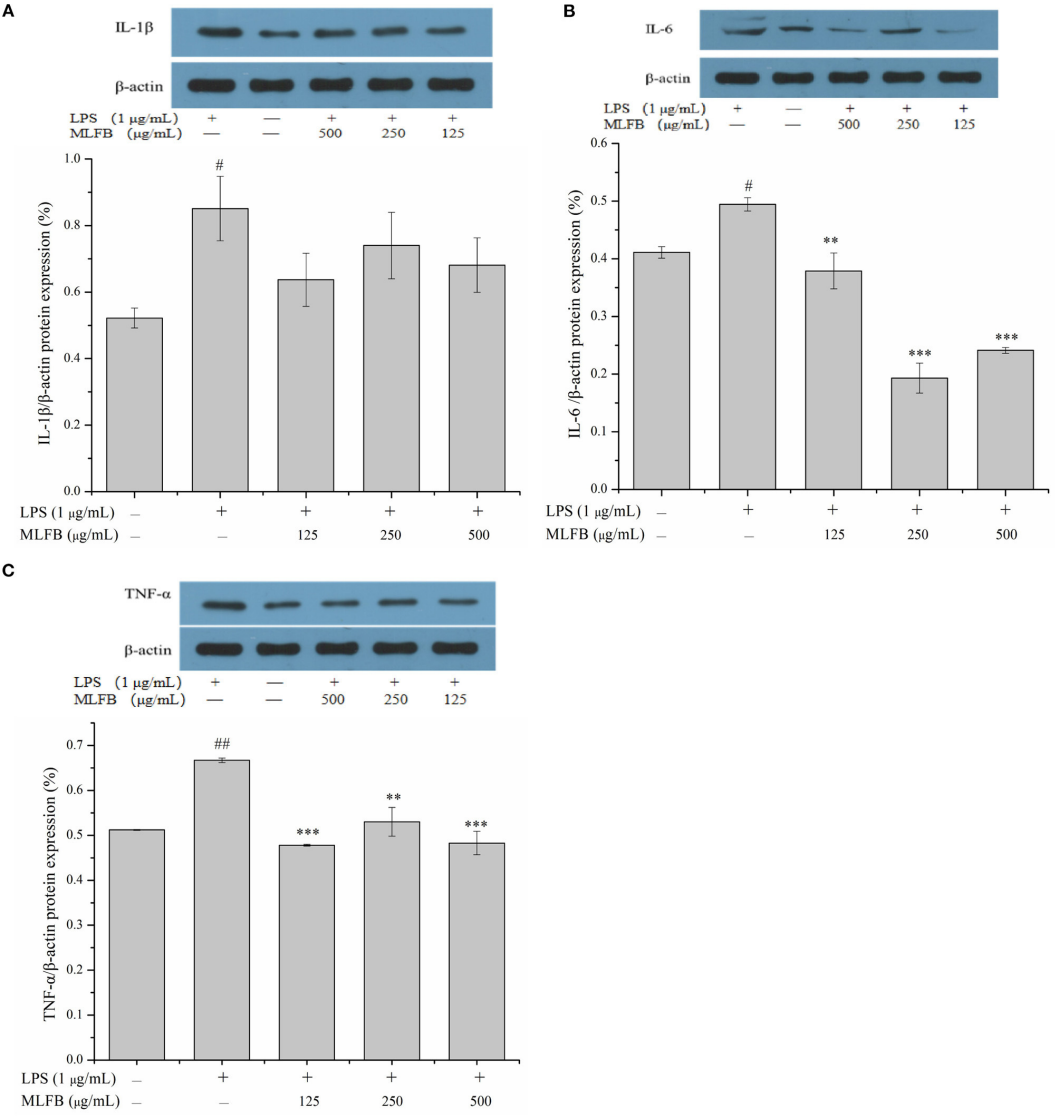
Effects of MLFB on the protein expressions of IL-1β **(A)**, IL-6 **(B)**, TNF-α **(C)** in LPS-induced RAW 264.7 cells. ^#^Means statistical difference compared with blank control group (^#^*P* < 0.05, ^##^*P* < 0.01). *Means statistical difference compared with LPS-induced inflammatory model group (**P < 0.01, ***P < 0.001).

IL-1β, IL-6, IL-8, and TNF-α are typical pro-inflammatory cytokines that secreted by activated macrophages, and are important in inflammatory responses (21). Dysregulation of these pro-inflammatory cytokines is related to systemic inflammatory disorder and may lead to various inflammatory diseases like rheumatoid arthritis and inflammatory bowel disease (21). The effects of MLFB on the productions, mRNA and protein expressions of LPS-induced pro-inflammatory cytokines including IL-1β, IL-6, IL-8 and TNF-α were determined by ELISA, RT-qPCR and Western blot, respectively. As shown in Figure 2, the productions of IL-1β, IL-6, IL-8 and TNF-α in LPS-induced inflammatory model group were obviously more than those in normal group, which indicated that the cellular inflammatory model was successfully established. Compared with the inflammatory model group, the productions of pro-inflammatory factors IL-1β, IL-6, IL-8 and TNF-α in cells were dose-dependently decreased after treated with a series concentrations of MLFB. When the concentration of MLFB reached 500 μg/mL, the productions of IL-1β, IL-6, IL-8 and TNF-α in the MLFB treatment groups decreased by 63.22, 35.71, 59.89, and 60.77%, respectively. The mRNA expression levels of pro-inflammatory cytokines IL-1β, IL-6 and TNF-α in RAW 264.7 cells were shown in Figure 3. As expected, the mRNA expressions of IL-1β, IL-6 and TNF-α in LPS-induced inflammatory model group were obviously increased when compared with normal group. Despite the treatment of MLFB at concentrations of 125 μg/mL showed no significantly inhabitation effects on the mRNA expression of these pro-inflammatory cytokines induced by LPS, when the concentrations of MLFB increased to 250 and 500 μg/mL, the mRNA expression of these pro-inflammatory cytokines were obviously inhibited. Similarly, compared with LPS-induced inflammation model group, the protein expression levels of pro-inflammatory factors IL-1β, IL-6, TNF-α were decreased in different degrees after treated with different concentrations of MLFB, despite the decrease of IL-1β protein expression was not significant (Figure 4).

The above results indicated that the MLFB could down-regulate the expressions of pro-inflammatory cytokines in macrophages activated by LPS, thus further inhibit the secretions of these pro-inflammatory cytokines and exerts anti-inflammatory effect. Previous studies have showed that GABA could promote proliferation of T-cells, inhibit apoptosis in β-cells, decrease the synthesis of inflammatory mediators like IL-1β, TNF-α, IFN-γ, IL-12, increase the production of anti-inflammatory mediator TGF-β1, and thus played important roles in anti-inflammatory responses (7, 8). Besides, the ability of GABA to restrain the secretions of TNF-α, COX-2 and iNOS, IL-6 and IL-12 by LPS-induced macrophages was also reported (22, 23). Therefore, the anti-inflammatory activity of MLFB may be attributed to its high content of GABA. Our study result is similar to the study result of Chang et al. (24) who reported that GABA-enriched pepino extract obtained after fermentation can inhibit the expression of TNF-α in LPS-induced RAW 264.7 macrophages.

### 3.3. MLFB inhibits the expressions of PGE_2_ and iNOS in LPS-induced RAW 264.7 cells

**Figure 5:**
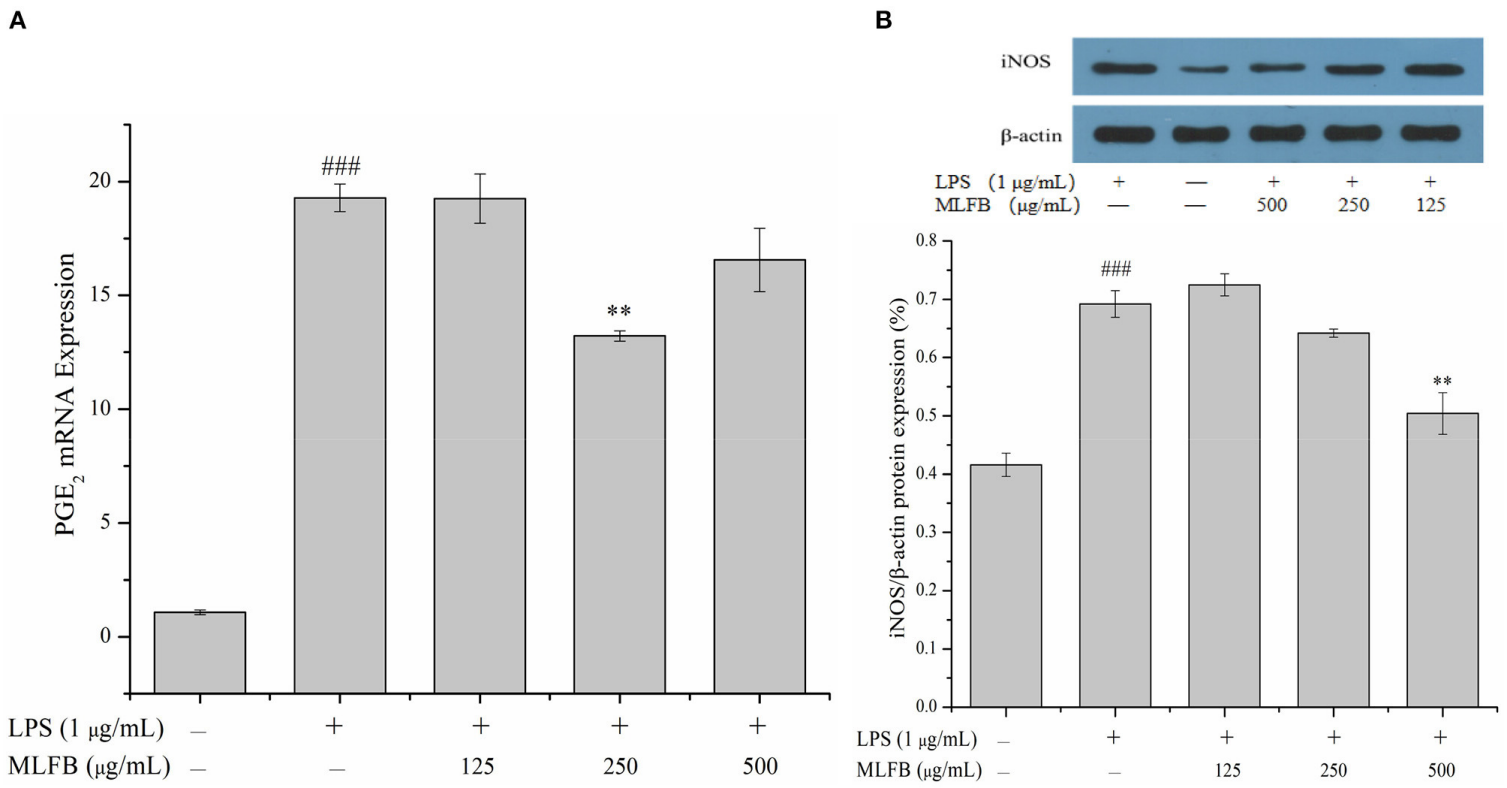
Effects of MLFB on the expressions of PGE_2_ **(A)** and iNOS **(B)** in LPS-induced RAW 264.7 cells. ^#^Means statistical difference compared with blank control group (^###^*P* < 0.001). *Means statistical difference compared with LPS-induced inflammatory model group (**P < 0.01).

Prostaglandin E_2_ (PGE_2_), an important prostanoid metabolite mainly produced by cyclooxygenase-2 (COX-2), is involved in multiple inflammatory processes including activating immune cell and promoting the secretion of pro-inflammatory cytokines (25). A variety of stimulations of inflammation or tissue injury will lead to overproduction of PGE_2_, thus inhibiting the expression of PGE_2_ is also an effective measure to treat inflammation (20). The effect of MLFB on the mRNA expression of PGE_2_ was shown in Figure 5A. The result showed that the mRNA expression of PGE_2_ in cells were significantly increased after treated with LPS, and 125 μg/mL of MLFB displayed no significant effect on the mRNA expression of PGE_2_ compared with LPS-treated group. Notably, the mRNA expression of PGE_2_ in cells treated with 250 μg/mL of MLFB was significantly decreased. The result indicated that MLFB at a certain dose could inhibit the expressions of PGE_2_ and therefore control the overproduction of PGE_2_ induced by LPS.

Inducible NOS (iNOS), an isoform of nitric oxide synthase (NOS), is especially expressed during inflammatory response and involved in the synthesis of pro-inflammatory mediator nitric oxide (NO) (25). The over expression of iNOS will produce excess amount of NO, thus leading to tissue damage, septic shock, and further resulting in other complication during persistent chronic inflammatory response (21). The effect of MLFB on the protein expression of iNOS in RAW 264.7 macrophages was exhibited in Figure 5B. The intracellular protein expression of iNOS in LPS-induced inflammatory model group was obviously higher than that in normal group. Compared with the inflammatory model group, the protein expression of iNOS in cells was dose-dependently decreased after treated with different concentrations of MLFB. The result indicated that MLFB could inhibit the expression of iNOS, which may further suppress the production of NO, thus alleviate LPS-induced inflammatory response.

COX-2 and iNOS are primary inflammatory mediators expressed in macrophages which are involved in the synthesis of PGE_2_ and NO, and the production of PGE_2_ and NO are closely related with initiation of the early stage of inflammatory pathways (1, 26). In the present study, pretreatment with MLFB effectively inhibited the mRNA expression of PGE_2_ and protein expression level of iNOS in LPS-stimulated RAW 264.7 macrophages at different degree, which indicated that MLFB involved in the suppression in the early stage of inflammatory pathways. Previous studies illuminated that ethyl acetate fraction, concentrate and isothiocyanates of *Moringa oleifera* could effectively down-regulate the expression of COX-2 and iNOS, and concomitantly inhibited the production of NO and PGE_2_ in LPS-stimulated RAW 264.7 macrophages (1, 13). In addition, GABA is also reported to have the ability of inhibiting immune cells activation by diminishing the production of COX-2 and iNOS (6). Therefore, the GABA as well as other active constituents contained in *Moringa oleifera* leaves may play roles in the inhibition of PGE_2_ and iNOS expressions.

### 3.4. MLFB suppresses inflammation mediated by the TLR-4/NF-κB signaling pathway

**Figure 6:**
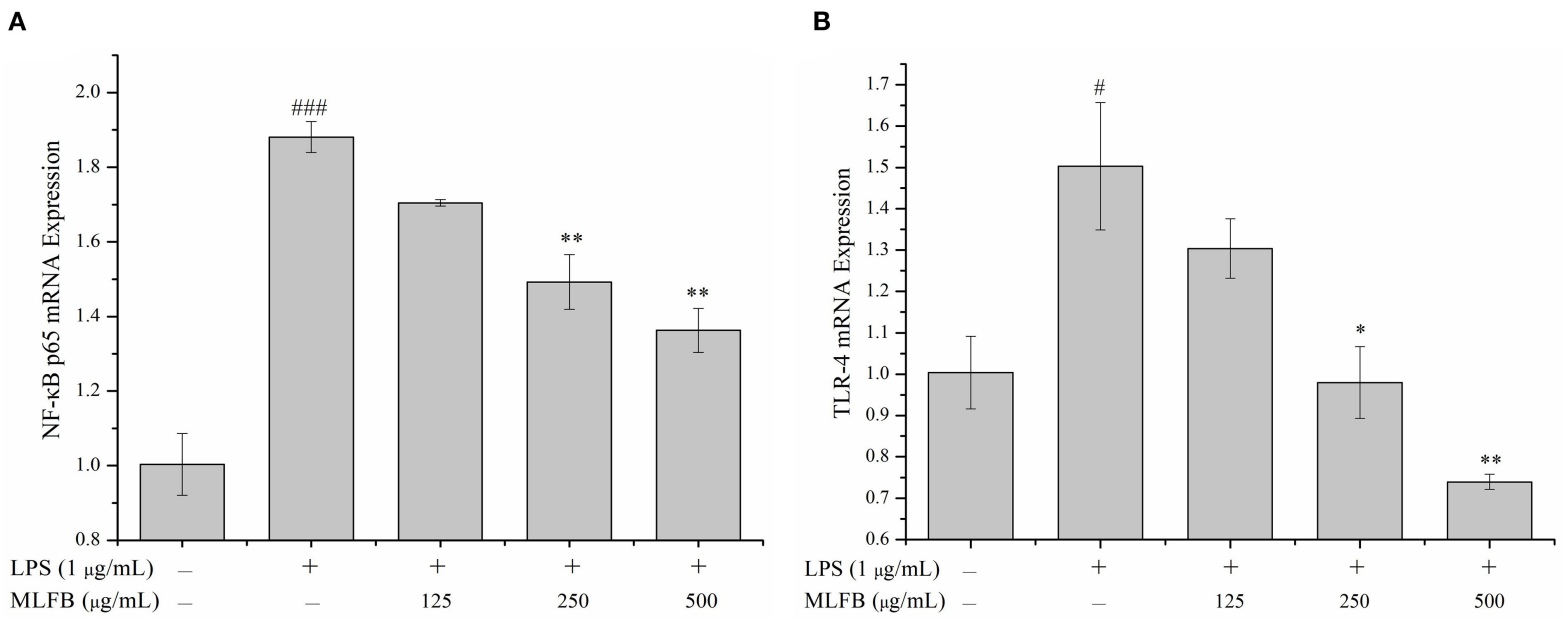
Effects of MLFB on the mRNA expressions of NF-κB p65 **(A)** and TLR-4 **(B)** in LPS-induced RAW 264.7 cells. ^#^Means statistical difference compared with blank control group (^#^*P* < 0.05, ^###^*P* < 0.001). *Means statistical difference compared with LPS-induced inflammatory model group (*P < 0.05, **P < 0.01).

Toll-like receptors (TLRs) are important transmembrane signaling receptors mediating signal transductions, and TLR-4 is one of the most studied receptors involved in inflammatory response, immune response and other processes (27, 28). It has been reported that LPS could interact with TLR-4 and following activate several intracellular signaling pathways including NF-κB signaling pathway (29, 30). NF-κB, mainly composed of p50 and p65 subunits, is a transcription factor that plays an important role in regulating innate and adaptive immunity, including inflammation signal transduction of macrophages (31, 32). In the normal state, NF-κB exists in cytosol and is combined with inhibitory protein IκB. When the cells were stimulated by LPS, IκB is phosphorylated and rapidly degraded by proteasomes in the cytoplasm, thus NF-κB is released (28). The free NF-κB transfers into the nucleus and turn on gene expression of pro-inflammatory mediators (33, 34). In a word, TLR-4/NF-κB signaling pathway participates in the regulations of various inflammatory cytokines like IL-1β, IL-6, IL-8, and TNF-α, and plays an important role in both immune and inflammatory response (8, 35). Interdicting the TLR/NF-κB signaling pathway will be an effective method for treatment of chronic inflammatory diseases. Therefore, the key molecules (TLR-4 and NF-κB p65) in the TLR-4/NF-κB signaling pathway were detected to explore the anti-inflammatory mechanism of MLFB on LPS-induced RAW 264.7 cells. As shown in Figure 6, comparing to the normal group, the mRNA expression level of NF-κB p65 and TLR-4 in RAW 264.7 cells were significantly increased after stimulated by LPS, indicating that the TLR-4/NF-κB signaling pathway was activated by LPS. When the cells were pretreated with different concentrations of MLFB, the mRNA expression levels of NF-κB p65 and TLR-4 in cells were obviously decreased in a dose-dependently manner. The result demonstrated that MLFB suppressed the mRNA expression levels of the key molecules in the NF-κB signaling pathway induced by LPS in RAW 264.7 cells. Therefore, MLFB may exert anti-inflammatory activity by inhibiting the TLR-4/NF-κB signaling pathway.

### 3.5. The key active components analysis of MLFB and its anti-inflammatory mechanism elaboration

The changes of key active components like total flavonoids, total polyphenols and organic acid content in MLFB after fermentation was shown in Table 2. After fermentation, the total flavonoids content decreased from 48.66 mg/L to 31.81 mg/L, while total polyphenols content increased from 21.43 mg/L to 26.02 mg/L, which was increased by 21.42% compared with the unfermented MLS. The content changes of flavonoids and polyphenols during fermentation may be due to their continuous degradation, convertation, and synthesis during the growth and metabolic processes of microorganisms (36). For instance, it is reported that microbial degradation may cause the cleavage and hydroxylation of the aromatic ring in flavonoids and polyphenols (37). Their precipitation or oxidation during the fermentation process may also lead to decrease of these compounds (38). Conversely, some lactobacillus like *L. rhamnosus* are reported to have the ability of releasing esterases which can hydrolyze ester bonds between phenolic acids and cell wall substances, thus resulting in the release of phenols (39). Previous studies also showed that lactobacillus can produce glycosyl hydrolases which can convert the bound phenolics linked with glycosides into free phenols during fermentation (40). As shown in Table 2, the contents of citric acid and succinic acid in MLFB decreased to < 1 mg/L after fermentation, but the content of pyruvic acid, lactic acid and acetic acid were increased obviously (*P* < 0.05). The content of lactic acid greatly increased to 303.84 mg/L and became the main organic acid in MLFB. However, the contents of oxalic acid showed no significantly changes after fermentation. The content changes of organic acids in MLFB may be associated with glycolytic pathway and tricarboxylic acid (TCA) cycle during the growth and metabolic processes of lactobacillus. During fermentation process, lactobacillus can catabolize sugars into lactic acid and acetic acid through the glycolytic pathway (38, 40). Besides, citric acid, pyruvic acid and succinic acid are important intermediate metabolites of the TCA cycle and the contents of these organic acid change dynamically with the involved biochemical reactions (40, 41). For example, citric acid is used as the second carbon source by lactobacillus and can be converted into acetic acid and oxaloacetic acid by a citrate lyase, while the oxaloacetic acid can be further translated into pyruvic acid which is eventually reduced to lactic acid by the lactate dehydrogenase (42). In addition, in the oxidative branch pathway of the TCA cycle, oxaloacetate can also be converted into succinic acid through a serious of enzymes like aconitase, isocitrate dehydrogenase, and succinic acid can be further converted to fumarate by succinate dehydrogenase (43). The changes of total polyphenols as well as citric acid, succinic acid, lactic acid and oxalic acid contents were similar to our previous study (17), in which the contents of polyphenols and lactic acid increased, citric acid and succinic acid decreased, and oxalic acid showed no obvious changes after the *Moringa oleifera* leaves were fermented with *Lactobacillus plantarum S35*. However, the changes of total flavonoids, pyruvic acid and acetic acid content in this study showed opposite trend with our previous study (17), which might due to the difference of fermentation condition and the used fermentation strain. Furthermore, in the study of Jin et al. (11), except for GABA content, the lactic acid and acetic acid contents in GABA-enriched green tea were also significantly increased after fermentation with *Levilactobacillus strain GTL 79*, which was similar with present study result.

**Table 2:** The key active components changes in MLFB after fermentation.

**Active components**	**MLS**	**MLFB**
Flavonoids (mg/L)	48.66 ± 3.93	31.81 ± 2.35^*^
Polyphenols (mg/L)	21.43 ± 1.48	26.02 ± 0.33^*^
**Organic acids**		
Oxalic acid (mg/L)	14.14 ± 0.70	13.71 ± 0.53
Citric acid (mg/L)	26.44 ± 0.39	< 1.00^*^
Pyruvic acid (mg/L)	4.28 ± 0.04	12.19 ± 0.52^*^
Succinic acid (mg/L)	19.16 ± 1.68	< 1.00^*^
Lactic acid (mg/L)	0	303.84 ± 8.13^*^
Acetic acid (mg/L)	0.81 ± 1.76	5.36 ± 0.08^*^

^*^Means statistical significant compared to MLS (*P* < 0.05).

It has been reported that GABA suppressed the mRNA expression levels of key mediators in the NF-κB signaling pathway induced by LPS in MAC-T cells, such as TLR-4, myeloid differentiation 88 (MyD88), NF-κB p65 and TNF receptor-associated factor 6 (TRAF6), which indicated that GABA might suppresses inflammation by inhibiting the activity of the NF-κB signaling pathway (8). Furthermore, previous studies also showed that *Moringa oleifera* extract pretreatment could block LPS-induced activation of NF-κB p65 subunit in MAC-T cells and thus protect bovine mammary epithelial cells against inflammation (35). Several reports indicated that the high content of flavonoids and phenolic compounds contained in *Moringa oleifera* leaves contributed to its anti-inflammatory activity (15, 44, 45). In addition, it has been reported that the enhanced effect of fermentation on the anti-inflammatory activity of *Laminaria japonica* was possibly due to the productions of more bioactive constituents after fermentation, such as fucoidan, alginate and phenols (20). Zhang et al. (3) also verified that the enhancement of antioxidant and anti-inflammatory activities of rape bee pollen after fermentation were mainly due to the increase of phenolics, flavonoids, fatty acids, and amino acids. Previous studies showed that except for the increase of GABA content, other active constituents like flavonoids, polyphenols, oligosaccharides, organic acids of *Moringa oleifera* leaves were also significantly increased after fermentation (12, 17). In our study, the GABA (18), total polyphenols, and most organic acids like pyruvic acid, lactic acid as well as acetic acid were increased obviously after fermentation. Though the total flavonoids content in MLFB decreased after fermentation, there were still 32 mg/L of flavonoids remained. Therefore, the anti-inflammatory activity that MLFB showed on LPS-induced cell inflammatory model might be associated with GABA and other active constituents such as flavonoids, phenolics and organic acids contained in the fermentation broth.

**Figure 7:**
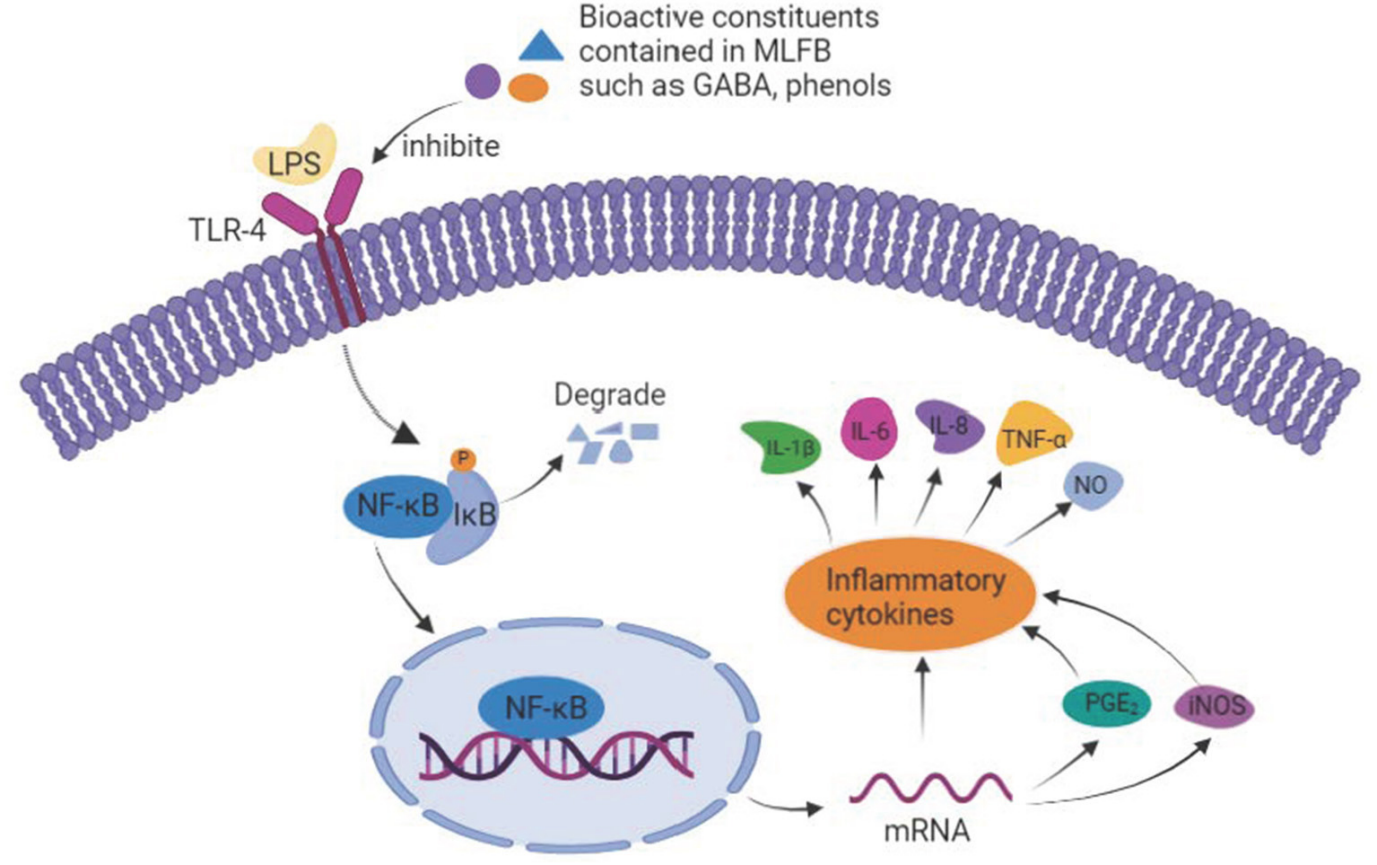
The underlying anti-inflammatory mechanism of MLFB.

The possible anti-inflammatory mechanism of MLFB was summarized in Figure 7. In detail, the high content of GABA and other active constituents like flavonoids, polyphenols contained in MLFB inhibited the expression of the main transmembrane signaling receptor TLR-4, and therefore disturbed its interaction with LPS. As a result, the stimulation of LPS to the macrophages was reduced and the NF-κB signaling pathway was inhibited, which further led to the secretion decrease of pro-inflammatory mediators and cytokines like iNOS, PGE_2_, IL-1β, IL-6, IL-8 and TNF-α, eventually the inflammation was alleviated.

## 4. Conclusion

In this study, the anti-inflammatory activity of MLFB was investigated and its underlying anti-inflammatory mechanism was clarified on LPS-induced RAW 264.7 cells model combining with its key active components analysis before and after fermentation. The results showed that MLFB suppressed the secretions and expressions of pro-inflammatory cytokines such as IL-1β, IL-6, IL-8 and TNF-α in LPS-induced RAW 264.7 cells. In addition, the expressions of PGE_2_ and iNOS were inhibited by MLFB, which might contribute to the decrease of pro-inflammatory cytokines and NO productions. Moreover, the mRNA expressions of the key molecules (TLR-4 and NF-κB p65) in the NF-κB signaling pathway were also restrained by MLFB in a dose-dependent manner, which indicated that the activity of the NF-κB signaling pathway was inhibited. In conclusion, MLFB can effectively ameliorate LPS-induced inflammation by inhibiting the secretions of pro-inflammatory cytokines and its underlying mechanism may be associated with the inhibition of TLR-4/NF-κB inflammatory signaling pathway activation. Besides, the anti-inflammatory activity of MLFB might related to the relative high contents of GABA as well as other active constituents such as flavonoids, phenolics and organic acids in MLFB. Our research provides a theoretical basis for the follow-up development of GABA-enriched *Moringa oleifera* functional foods with anti-inflammatory functions. However, the present study about the anti-inflammatory mechanism of MLFB was preliminary. Its in-depth mechanisms against inflammation should be further clarified and the active ingredients contained in MLFB also need to be further clarified.

## Data availability statement

The original contributions presented in the study are included in the article/Supplementary material, further inquiries can be directed to the corresponding authors.

## Author contributions

LZ: conceptualization, methodology, and validation. XL: data curation, writing—original draft preparation, and visualization. SY: methodology and data curation. FZ: funding acquisition and validation. YZ: resources and writing—review and editing. SX: formal analysis and validation. YC: conceptualization and funding acquisition. WZ: writing—review and editing, funding acquisition, and supervision. All authors contributed to the article and approved the submitted version.
