# Supplementary material for: The anti-inflammatory activity of GABA-enriched Moringa oleifera leaves produced by fermentation with Lactobacillus plantarum LK-1
Source: Front Nutr. 2023 Mar 9;10:1093036. doi: 10.3389/fnut.2023.1093036 (PMC10034114; doi:10.3389/fnut.2023.1093036)
Supplement: Supplementary file 1 [file Image_1.PDF]

Appendix

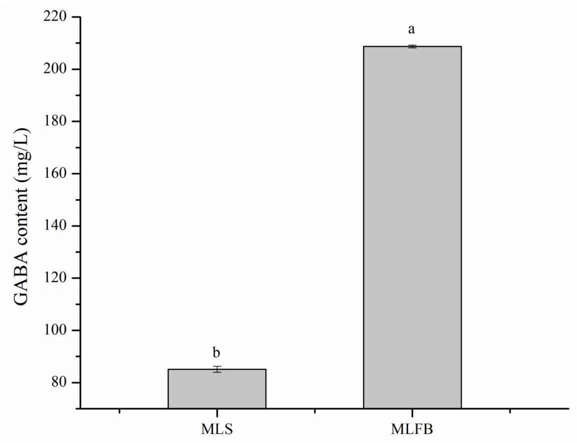

Figure 1. The GABA contents of *Moringa oleifera* leaves solution (MLS) and GABA-enriched *Moringa oleifera* leaves fermentation broth (MLFB). Different lower case letters at the top of the bars denote significant differences ($P < 0.05$).

(A)

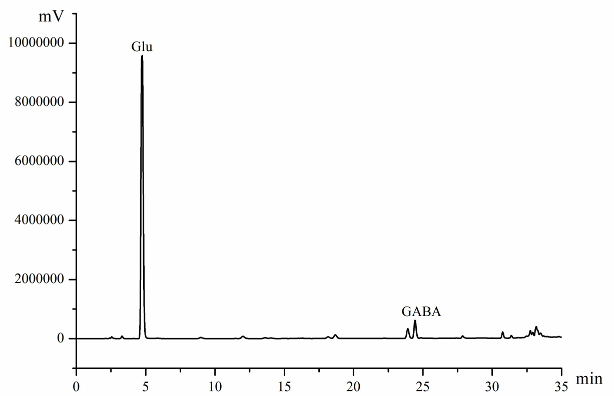

(B)

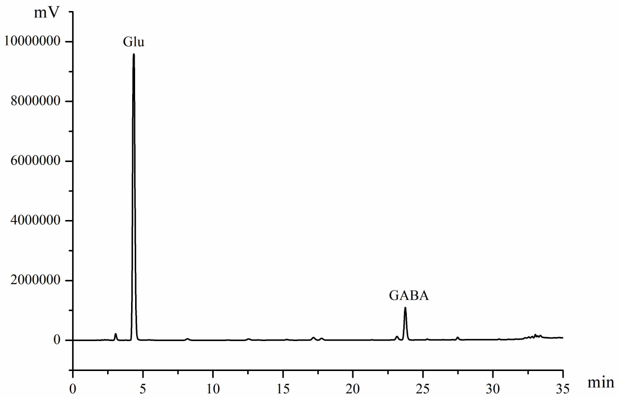

Figure 2. The HPLC spectrograms of *Moringa oleifera* leaves solution (MLS) and GABA-enriched *Moringa oleifera* leaves fermentation broth (MLFB) about GABA content analysis.
